# Independent Evolution of Transcriptional Inactivation on Sex Chromosomes in Birds and Mammals

**DOI:** 10.1371/journal.pgen.1003635

**Published:** 2013-07-18

**Authors:** Alexandra M. Livernois, Shafagh A. Waters, Janine E. Deakin, Jennifer A. Marshall Graves, Paul D. Waters

**Affiliations:** 1 Evolution, Ecology and Genetics, Research School of Biology, The Australian National University, Canberra, Australian Capital Territory, Australia; 2 School of Biotechnology & Biomolecular Sciences, Faculty of Science, University of New South Wales, Sydney, New South Wales, Australia; 3 La Trobe Institute of Molecular Sciences, La Trobe University, Melbourne, Victoria, Australia; University of California Los Angeles, United States of America

## Abstract

X chromosome inactivation in eutherian mammals has been thought to be tightly controlled, as expected from a mechanism that compensates for the different dosage of X-borne genes in XX females and XY males. However, many X genes escape inactivation in humans, inactivation of the X in marsupials is partial, and the unrelated sex chromosomes of monotreme mammals have incomplete and gene-specific inactivation of X-linked genes. The bird ZW sex chromosome system represents a third independently evolved amniote sex chromosome system with dosage compensation, albeit partial and gene-specific, via an unknown mechanism (i.e. upregulation of the single Z in females, down regulation of one or both Zs in males, or a combination). We used RNA-fluorescent *in situ* hybridization (RNA-FISH) to demonstrate, on individual fibroblast cells, inactivation of 11 genes on the chicken Z and 28 genes on the X chromosomes of platypus. Each gene displayed a reproducible frequency of 1Z/1X-active and 2Z/2X-active cells in the homogametic sex. Our results indicate that the probability of inactivation is controlled on a gene-by-gene basis (or small domains) on the chicken Z and platypus X chromosomes. This regulatory mechanism must have been exapted independently to the non-homologous sex chromosomes in birds and mammals in response to an over-expressed Z or X in the homogametic sex, highlighting the universal importance that (at least partial) silencing plays in the evolution on amniote dosage compensation and, therefore, the differentiation of sex chromosomes.

## Author Summary

Dosage compensation is a mechanism that restores the expression of X chromosome genes back to their original level when Y homologues lose function. In placental and marsupial mammals this is achieved by upregulating the single X in males. The carry-through of overexpression to females would result in functional tetraploidy, so there is subsequent inactivation of one X chromosome in the somatic cells of females, leaving males (XY) and females (XX) with a single upregulated X. In contrast, genes on the five platypus (a monotreme mammal) X chromosomes and the chicken Z chromosome (which are orthologous but independently evolved) are expressed globally at a higher level in female platypus and male chicken respectively, indicating partial dosage compensation. Here, for the first time, we provide evidence for inactivation of genes on the chicken Z chromosome in ZZ males, and on all five Xs in female platypus. Our results suggest that the silencing of genes on sex chromosomes has evolved independently in birds and mammals, and is, therefore, a critical step in the pathway to dosage compensate independently evolved amniote sex chromosomes systems.

## Introduction

Vertebrates with heteromorphic sex chromosomes have either male heterogamety like humans (XX female and XY male), or female heterogamety like birds (ZZ male and ZW female). Degeneration of the non-recombining Y or W chromosome, central to the evolution of sex chromosomes, left genes on the X or Z as a single copy in the heterogametic sex. This resulted in an imbalance of X or Z gene dosage relative to the autosomes, and between the sexes.

Different dosage compensation systems have arisen independently in diverse organisms, suggesting that dosage compensation is critical for the survival of species with differentiated sex chromosomes. Ohno [1] hypothesized that degeneration of the Y/W chromosome would result in under expression from the X/Z in the heterogametic sex (equivalent to monosomy), which would result in pressure to up regulate the single X/Z to restore parity with the autosomes. Because the homogametic sex has two X/Z chromosomes, over expression would result in doubled normal expression (equivalent to tetrasomy), which would result in pressure for global down regulation of the X/Z to again resort parity with the autosome (reviewed in [2]). However, recent data has questioned global over regulation of the X/Z [3], [4], sparking considerable debate [5]–[7] and suggestion that dosage compensation evolved in response to a subset of dosage sensitive genes [8].

In eutherian mammals one whole X is transcriptionally silenced in the somatic cells of females, although many genes located on the evolutionarily more recent region escape silencing [9]. X inactivation (XCI) is established early in embryogenesis and is somatically heritable. In eutherian mammals the silenced X is chosen at random in a process governed by a large non-coding RNA transcribed from a locus called *XIST* (X-inactive-specific transcript) [10]–[12]. In all eutherian mammals, *XIST* is transcribed from the X to be inactivated, which it coats *in cis* during early development, although the timing and regulation of expression varies between species [13]. After one X is chosen for inactivation, a specific signature of epigenetic modifications is established [14], which appears to be conserved in even the most distantly related eutherians [15].

The increasing availability of genomic data now makes it possible to study dosage compensation in non-traditional model organisms such as marsupial and monotreme mammals. In marsupials, XCI is imprinted, with the paternal X always being the inactivated homologue [16]. X-inactivation in representative Australian marsupials is thought to be incomplete, tissue-specific and gene-specific [17]. RNA-fluorescent *in situ* hybridization (RNA-FISH) showed that within a population of fibroblasts cells derived from female tammar wallaby there was a mixture of 1X-active and 2X-active nuclei, the proportion of which was characteristic of each locus [18]. A similar profile was obtained for human and elephant X genes that escape inactivation [19]. However, it was shown (with RNA-FISH) in post-mortem tissue of the South American grey short-tailed opossum that X-inactivation is efficient [20], and RNA-sequencing revealed that X expression levels are similar between the sexes [21].

The egg-laying monotreme mammals (platypus and echidna species) have multiple sex chromosomes that share homology, not with the therian X, but the bird Z [22], [23]. Recent transcriptome sequencing in platypus showed that compensation is achieved via upregulation of X genes in males, and that global X-inactivation in females is likely unnecessary [21]. However, as for marsupials and escaper genes on the human X, RNA-FISH showed that within a population of platypus fibroblast cells, there was a mixture of 1X-active and 2X-active cells, each locus having a characteristic frequency of inactivation [24].

Data on dosage compensation in birds are fragmentary and contradictory. Real-time PCR showed equivalent expression of most Z-borne genes in ZZ male and ZW female chick embryos (that is, complete compensation) for most genes [25], but global microarray analysis in chicken, and a small cDNA microarray in zebra finch showed that male to female ratios were significantly higher for Z genes than for autosomal genes [26]. A male: female Z gene dosage of approximately 1.5 was demonstrated by microarray [27] and RNA-sequencing [21] analyses in chicken, and dosage ratios of 1.23 was observed in zebra finch [28] and 1.36 in crow [29]. The incomplete dosage compensation of Z-linked genes, at least in chicken, was reported to be regulated locally on a gene-by-gene basis, and is tissue and developmental stage specific [30].

It is difficult to compare dosage compensation between birds and mammals. However, recent comparative transcriptome sequencing [21] indicated that genes on the single Z in female chicken had equivalent expression levels to orthologous genes in outgroup species, where the Z is autosomal (i.e. expression from one Z equals expression from two proto-Zs), providing evidence for global upregulation of the single Z in females. In male chicken (with two Zs) the ZZ:proto-ZZ expression ratio was 1.13 to 1.56, indicating that Z gene upregulation was not specific to females. A similar pattern, although less clear, was observed for genes on platypus X_5_. Less efficient upregulation in the homogametic sex (i.e. ZZ/X_5_X_5_: ZW/X_5_Y_5_ expression ratio <2) indicated that upregulation was more efficient in the heterogametic sex, or that there was a mechanism to partially reduce expression of Z/X_5_ in the homogametic sex. Julien et al. [21] suggested that dosage compensation only mildly affects the homogametic sex in platypus and chicken and, as such, there was potentially no requirement for the evolution of Z/X_5_ inactivation.

Here we use RNA-FISH to examined chicken and platypus dosage compensation, which permits detection of transcription from one or both alleles at specific loci in individual nuclei. We found that genes on the chicken Z, as well as on the partially homologous platypus Xs, were expressed from one (or both) alleles in characteristic frequencies for different loci, just as on the independently evolved therian X. Our results indicate that silencing mechanisms were exapted multiple times in the homogametic sex (likely in response to the upregulation of genes on the Z/X in the heterogametic sex) resulting in transcriptional inactivation on non-homologous Zs and Xs in distantly related species.

## Results

We examined transcription in individual fibroblasts, using RNA-FISH. As probes, we chose bacterial artificial chromosome (BAC) clones containing known genes on the Z chromosome in a representative bird (chicken), and on the X chromosomes in a representative monotreme (platypus). For both species there is a genome assembly available [31], [32].

### Partial Inactivation of Z Genes in Chicken Cells

As controls, we chose ten BACs containing known autosomal genes, including a BAC containing *GAPDH*, which was used as a control in all experiments. In RNA-FISH experiments with each of the ten autosomal BACs, we observed two signals in at least 97% of nuclei in both male and female fibroblast cells (Table S1 and Figure S1). Thus, autosomal genes are generally transcribed from both alleles in cultured chicken fibroblasts.

We selected eleven Z-borne BACs, five of which contained a single gene, and six contained two or more genes (Table S1). We performed two-color RNA-FISH, with the autosomal control (*GAPDH*) and each test gene (Z-borne and autosomal), on male (ZZ) and female (ZW) fibroblasts (Table S1), and scored ≥100 cells for each hybridization. To control for polyploidy and accessibility of the probe into an individual nucleus, only nuclei with two signals from the control BAC were scored for the test BAC.

The frequency with which expression of the single Z in ZW females was detected as a single signal, controlled for the hybridization efficiency of each Z probe. All Z BAC probes displayed hybridization efficiencies of between 95–100% (Table S1). Even at the lowest hybridization efficiency of 95% (p = 0.95; q = 0.05), hybridization in ZZ male cells would produce few nuclei with 0 (q^2^ = 0.25%) or 1 (2pq = 9.5%) signal (see Materials and Methods).

**Figure 1:**
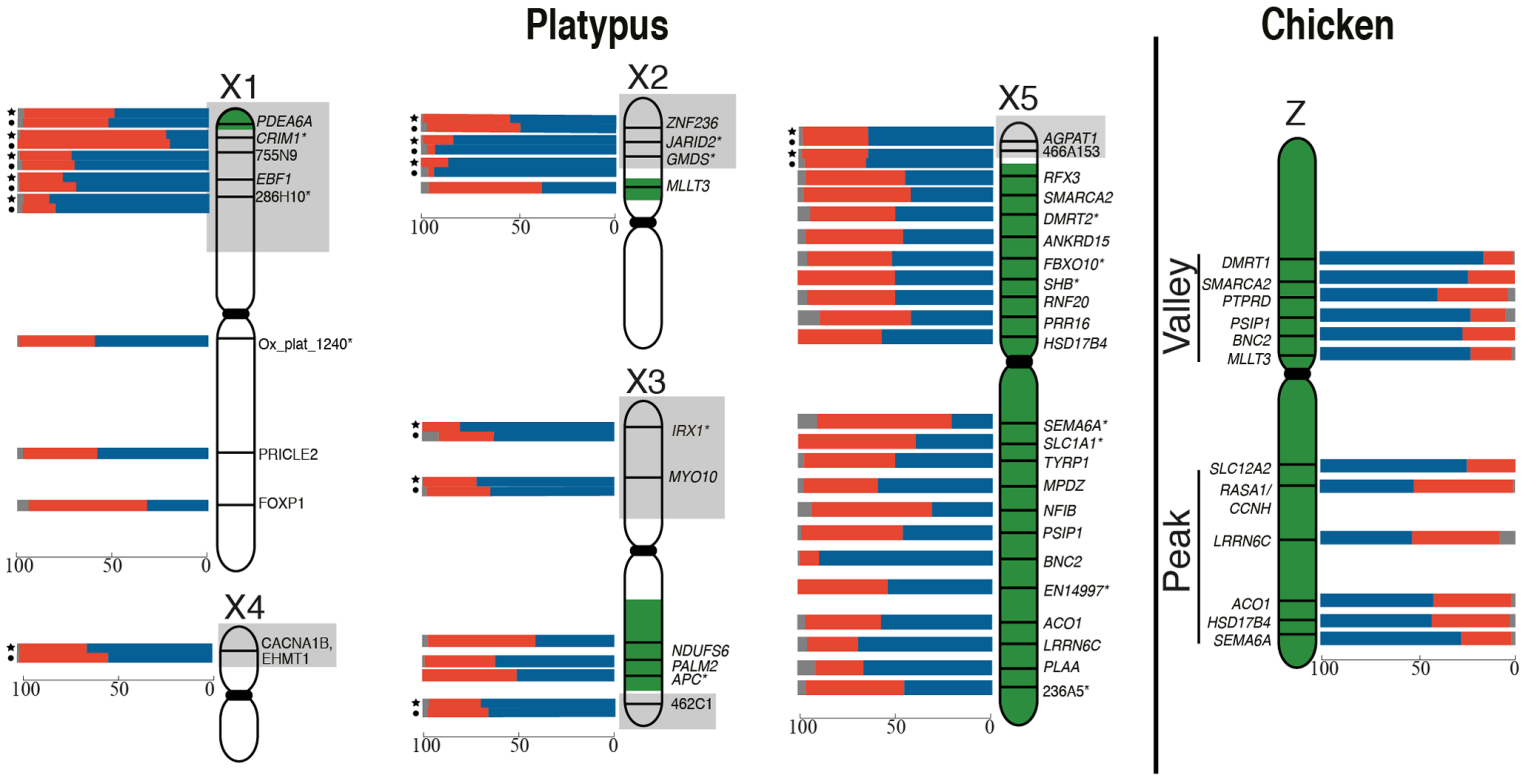
RNA-FISH activity maps of the platypus Xs and chicken Z chromosomes. Bars represent the percentage of homogametic nuclei transcribing 2 (blue), 1 (red) or 0 (grey) alleles for each locus. Loci in pseudoautosomal regions (grey boxes) were tested in both male (indicated by a circle) and female (indicated by a star). Green coloring on platypus Xs represents homology to the chicken Z [23]. Platypus X chromosomes are not to scale (see Figure S5). Genes denoted by * were analysed in [24].

For all probes hybridized to ZZ male nuclei, the cell population consisted of a mixture of nuclei that were 1Z-active (one signal) and 2Z-active (two signals) (Table S1). No locus was completely 1Z-active or completely 2Z-active. Instead, each Z locus was inactivated in a characteristic frequency of cells, ranging from 15% to 51% (Figure 1), that was significantly different (p<0.01 after Bonferroni correction and estimating experimental error; see Materials and Methods) from the number of 1-Z active nuclei expected due to inefficient hybridization. This frequency was reproducible between biological replicates for a subset of BACs, in which RNA-FISH was repeated on fibroblasts from a second individual (Table S1). The activity status of Z loci in a given nucleus did not appear to be clonally inherited (i.e. if the mother cell was 2Z-active both daughter cells should be 2Z-active; or conversely if the mother cell was 1Z-active both daughter cells should be 1Z-active); we observed daughter cells in our preparations in which one was 1Z-active and the other 2Z-active (Figure S2).

There was no obvious clustering of loci on the Z with particularly high or particularly low frequencies of 1Z-active cells, suggesting that the probability of transcriptional inhibition of a gene is independent of its physical location. The frequency of 1Z-active nuclei for the six BACs within the proposed dosage compensation ‘valley’ (129B9, 110A9, 164N4, 87K13, 57B13, and 89C2) was not lower than the five BACs in the ‘peak’ (65D18, 30H20, 73F14, 112C1, and 163I20) regions of the Z chromosome identified in chicken [28], [33]. However, there did appear to be a correlation of M∶F expression ratio (calculated from data in [34]; see Materials and Methods) of Z-genes, with the proportion of 1Z-active nuclei in males (R^2^ = 0.47, p = 0.03). If a gene was over expressed in males compared to females, there was a greater percentage of 1Z-active nuclei for that locus in males (Figure S3A).

**Figure 2:**
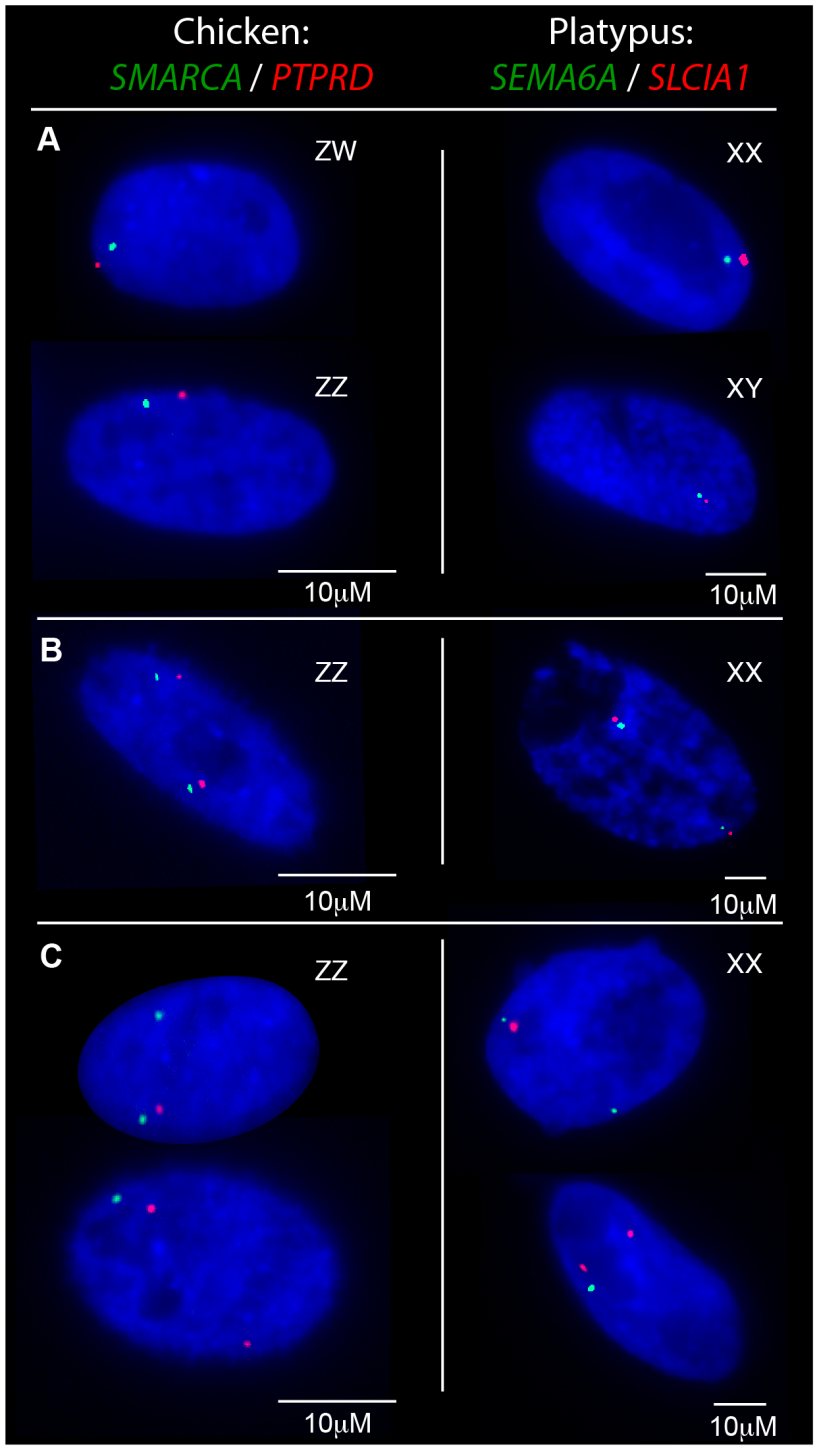
Transcriptional activity of neighbouring chicken Z loci and platypus X_5_ loci in fibroblasts. Gene names are color coded to correspond to signal color. **A**) In nuclei with only one allele active for both genes the signals co-locate in both sexes. **B**) Nuclei from the homogametic sex in which both genes are 2Z/2X-active. **C**) Nuclei from the homogametic sex in which the active Z/X expresses both genes and the other (inactivatable) Z/X expresses only one gene.

From these initial RNA-FISH results it could not be determine if transcriptional inhibition of different loci were on the same Z chromosome, or on different Z chromosomes. Therefore we used two-color RNA-FISH to examine transcription of two pairs of neighbouring Z genes in female and male chicken nuclei: *SMARCA2*/*PTPRD* (∼2 Mb apart), and *BNC2*/*MLLT3* (∼1.5 Mb apart) (Table 1) (Figure 2). In ZW female nuclei, we observed co-location (close proximity) of signals in 96% of nuclei that expressed both genes. In male nuclei, for each cell in which both loci were 1Z-active, we observed co-location of signals in 96% of nuclei (Figure 2A), indicating transcription from the same Z chromosome. This is consistent with the presence of an active Z (Za), and an inactive Z (Zi) on which genes are prone to silencing.

**Table 1:** RNA-FISH analysis of transcription from neighbouring loci in homogametic nuclei.

Gene Pair	Distance apart (Mb)	Number of nuclei scored	% nuclei with coordinate transcription
**Chicken Z**			
*SMARCA2*,*PTPRD*	2	98	96
*BNC2*, *MLLT3*	1.5	100	96
**Platypus X_5_**			
*MPDZ*, *NFIB*	0.5	30	100
*HSD17B4*, *SEMA6A*	1.8	26	100
*SLC1A1*, *SEMA6A*	0.5	22	100

Two-color RNA-FISH experiments with the same gene pairs also provided the opportunity to determine whether the silencing of neighbouring genes on the Zi chromosome was coordinated. In cells in which at least one of the neighbouring gene pairs was 2Z-active, we observed (for both Z gene pairs) that the second locus was simultaneously transcribed (i.e. both loci were 2Z-active in the same nucleus; Figure 2B–C) at a frequency no greater than expected by chance (see Materials and Methods; Table 2), consistent with independent silencing of tightly linked genes on Zi. The frequency of nuclei, in which both loci in a gene pair are expected to be 2Z- active, was the product of 2Z-active frequencies of each gene (from initial RNA-FISH results; Table S1).

**Table 2:** Frequency of nuclei transcribing both alleles of neighbouring loci.

Gene Pair	Number of nuclei scored	Expected (%)	Observed (%)	p-value
**Chicken Z**				
*SMARCA2/PTPRD*	216	45.6	45.67	0.201
*BNC2/MLLT3*	216	56.21	55.95	0.776
**Platypus X_5_**				
*MPDZ/NFIB*	243	18.6	55.87	<0.01
*HSD17B4/SEMA6A*	213	11.4	56.15	<0.01
*SLC1A1/SEMA6A*	170	7.8	52.05	<0.01

Expected frequencies of nuclei transcribing from both allelels of neighbouring loci were calculated from observed frequencies of 2Z-active (or 2X-active) nuclei (Table S1 and S2). For example, *SMARCA2* was 2Z-active in 76% of nuclei and *PTPRD* was 2Z-active in 60% of nuclei. Therefore, it was expected that they should both be 2Z-active in 0.76*0.6 (45.6%) of nuclei. P-values were calculated with a X^2^ test with 1 degree of freedom. Bonferroni correction was conducted.

### Partial Inactivation of X Genes in Platypus Cells

A total of 40 platypus BACs were analyzed: 19 in X-specific regions (two on X_1_, one on X_2_, two on X_3_, 14 on X_5_), nine in pseudoautosomal regions (PAR), and 12 on autosomes. The autosomal BAC bearing *HPRT* (on chromosome 6) was used as the autosomal control in all experiments. Hybridization efficiencies for each probe were assessed in male cells. These ranged from 94% to 100% (Table S2). At least 100 cells were scored for each hybridization.

In RNA-FISH experiments with each of the 12 autosomal BACs, we observed two signals in at least 95% of nuclei in both male and female fibroblast cells (Table S2 and Figure S4). Thus autosomal genes are generally transcribed from both alleles in cultured platypus fibroblasts.

For all X specific loci we observed 1X-active female nuclei at a significantly greater frequency (p<0.01 after Bonferroni correction and estimating experimental error; see Materials and Methods) than expected from inefficient hybridization. No loci were completely 1X-active, or 2X-active in every nucleus, and frequencies of 1X-active nuclei ranged from 25% to 62% for different loci. There was no obvious clustering of genes on any X with particularly high, or particularly low frequencies of 1X-active nuclei (Figure 1). However, for loci that were expressed at a higher level in females compared to males, there did appear to be a correlation of F∶M expression ratio (in cultured fibroblast calculated from data in [21]; see Materials and Methods) of X-genes with the proportion of 1X-active nuclei in females (R^2^ = 0.54, p = 0.004). If a gene was over expressed in females compared to males, there was a greater percentage of 1X-active nuclei for that locus in females (Figure S3B).

The activity status of X loci in a given nucleus was not strictly clonally inherited (i.e. if the mother cell is 2× active both daughter cell should be 2× active; or conversely if the mother cell is 1× active both daughter cell should be 1× active); we observed daughter cells in which one was 1X-active and the other 2X-active (Figure S2). We therefore conclude that genes on the platypus X chromosomes are subject to inactivation in a proportion of nuclei that is characteristic for each gene.

To determine if transcriptional inhibition of adjacent loci was coordinated, we used two-color RNA-FISH to examine transcription of three pairs of neighboring genes on platypus X_5_: *MPDZ*/*NFIB* (∼500 kb apart), *HSD17B4*/*SEMA6A* (∼1.8 Mb apart) and *SEMA6A*/*SLC1A1* (∼500 kb apart) (Table 1) (Figure 2A–C). As expected, in male controls we observed co-location of signals in 100% of nuclei for each gene pair. In female nuclei in which both loci were 1X-active, we also observed co-location of signals in 100% of nuclei for each gene pair tested (Figure 2A), consistent with the presence of a single active X_5_ (X_5_a), and an X_5_ (X_5_i) on which genes are prone to silencing.

Subsequently, we tested whether inactivation of adjacent loci was synchronized on X_5_i for these same neighbouring gene pairs by observing, in cells with one gene 2X-active, the frequency at which the second gene was 2X-active. We observed 2X-active nuclei at frequencies that were significantly (p<0.01) greater than expected by chance (Table 2, Figure 2; see Materials and Methods), indicating at least regional coordination of transcription on X_5_i. It was not possible to assess coordinated transcription over extended regions because FISH signals derived from X_5_a could not be distinguished from signals derived from X_5_i (see Materials and Methods).

Using RNA-FISH we also examined, in both male and female fibroblasts, transcription from genes located in the PAR of platypus sex chromosomes. We studied one locus in each of PARs X_2_/Y_2_, X_3_/Y_2_, X_3_/Y_3_ and X_4_/Y_3_, two loci in X_5_/Y_4_, and three loci in X_1_/Y_1_ (Figure S5). We expected to see nearly 100% 2X- active nuclei in both males and females, as for loci in the human PAR1, and the autosomal controls. However, all nine pseudoautosomal BACs tested were 1× active in 27–42% of male nuclei, and 16–47% of female nuclei, indicating significant (p<0.01 after Bonferroni correction) PAR gene inactivation in both sexes for seven of the nine loci tested (Table S2, Figure 1).

Importantly, two chicken autosomal BACs, orthologous to the platypus PAR genes that are subject to partial silencing, were 2-allele-active. Additionally, we demonstrated that four of the partially inactivated chicken Z/platypus X_5_ loci tested here are always 2 allele-active in human fibroblasts (Table S3), where they are autosomal. Finally, two of the biallelically expressed autosomal chicken BACs (Table S1), and four of the biallelically expressed autosomal platypus BACs (Table S2), tested here contained genes orthologous to X genes in human where they are subject to silencing. These experiments demonstrate that inactivation was dependent on the type of chromosome a locus was located on (i.e. sex chromosome or autosome), rather than it being a phenomenon unique to the loci examined here.

## Discussion

### Partial Sex Chromosome Inactivation

Using RNA-FISH, we demonstrate for the first time that Z inactivation plays a role in chicken dosage compensation, and confirmed that inactivation is also a feature of orthologous platypus X loci, as was previously shown by Deakin et al. [24].

Our observations conflict with those of a previous study of five chicken Z loci using RNA-FISH. Kuroda et al. [35] reported that most male nuclei expressed both Z alleles in five different tissue types (two Z loci were tested in liver, and one each in kidney, spleen and retina), and suggested that there was no inactivation on the chicken Z chromosome. The inconsistency in findings with this study may be due to the different tissue types used, and different inactivation profiles of chicken Z genes in other tissue types would not be surprising. We used fibroblast cells because they are easily collected and cultured from species that are difficult to sample, and to be consistent with our previous studies of sex chromosome silencing in mammals [18], [19], [24].

Additionally, Kuroda et al. [35] concluded that inactivation does not occur on the chicken Z because the frequency of detecting two signals of hybridization was similar for the autosomal probe and the Z probes (70–80%). However, the detection frequencies (1 or 2 signals observed) in male nuclei for the autosomal and Z probes were low (which were 66–77%, and were not co-hybridized). In this study we co-hybridized our autosomal control and test genes using one cell type, and only cells with two autosomal signals were scored for the test gene, controlling for ploidy and probe accessibility into individual nuclei. As a result we obtained much higher hybridization efficiencies, providing for much simpler interpretation.

An exhaustive study of male-female expression ratios using microarrays showed that different loci on the chicken Z are compensated to different extents [26], [30], and Julien et al. [21] eloquently demonstrated that the Z/X_5_ is upregulated in the heterogametic sex in chicken/platypus. These studies assessed transcription across whole cell populations, so could not distinguish between partial expression in all nuclei, and heterogeneity of expression of different cells. In male chicken fibroblast nuclei, we found for all loci a mixture of 1Z-active and 2Z active cells at reproducible frequencies (15% to 51% 1Z-active, depending on the locus). Therefore, we demonstrate that in the homogametic sex there is partial inactivation of one Z allele.

As such, these results demonstrate that an integral step of the partial bird dosage compensation system must include the silencing of one Z allele in males, analogous to the inactivation we observed here (and previously [24]) on the orthologous (although independently evolved) platypus X_5_, and the independently evolved therian mammal X chromosome. We also observe that loci over expressed in the homogametic sex were more likely to be 1Z/X-active, indicating that there is pressure to reduce expression of these genes in the homogametic sex. We conclude that partial inactivation on the Z in birds, and at least on four of the five X chromosomes in platypus, is likely in response to Z/X upregulation (perhaps of only several dosage sensitive genes) carrying through to the homogametic sex. Because higher transcript levels in the homogametic sex appear to correlate with a greater percentage of 1Z/X-active nuclei, evolution to down regulate the chicken Z (and platypus Xs) is conceivably an ongoing process. Were everything in equilibrium 1Z/1X-activity should correlate with a 1∶1 transcript ratio between the sexes.

### Distribution of Inactivation over the Sex Chromosome

We found no correlation between the extent of inactivation and the location of the gene on the chicken Z (Figure 1), and did not observe stronger inactivation of loci in the dosage compensation valley, which was detected on the chicken Z chromosome using microarrays [26], [28], [33], and is differentially methylated on the two Z chromosomes in males [36]. The functional significance of this dosage compensation valley is unclear because it is not conserved in zebra finch [28]. Other studies of chicken concluded that the dosage compensated valley region, concentrated in genes with low male to female expression ratios, simply contains genes with a strong female bias [30], [37], although this interpretation has also been challenged [38], [39]. It is important to note that our data does not necessarily conflict with original reports on the dosage compensation valley region [33], in which it was suggested that females up-regulate genes, rather than males down-regulating genes.

We also observed no clustering of platypus X-borne genes with very low or very high frequencies of 1X-active nuclei (Figure 1). This implies that the probability of X inactivation is not correlated with gene location, and provides no evidence for an X chromosome inactivation center from which inactivation might spread.

### Coordinate Control of Sex Chromosome Activity

We observed that adjacent genes were always expressed from the same Za chromosome in male chicken cells. This is consistent with a hypothesis that all loci on one Z chromosome are active (Za), and loci on the other Z (Zi) are prone to inactivation in a proportion of cells. We found that the probabilities of expression of adjacent loci on the Zi were not correlated, suggesting that escape from inactivation of each locus on Zi is independently regulated.

In female platypus cells, our analysis of three platypus X_5_ pairs showed co-location of signals for neighbouring loci (Table 2; Figure 2A), consistent with coordinate activity on one X_5_ (X_5_a) and inactivation of loci on the other X_5_ (X_5_i) in a proportion of cells in females. Partial activity of genes on Xi is consistent with the previous demonstration that both alleles were transcribed [24]. Unlike chicken, neighbouring genes on platypus X_5_ showed concordant 2X-activity (Figure 2B), implying that the regulation of transcription from X_5_i is at least under regional control.

We suggest, therefore, that in cells from male chickens there is one active Za, and one partially inactive (“inactivatable”) Zi on which loci have specific probabilities of being inactivated. Likewise, we propose that in cells from female platypus, there is one active Xa and one partially inactive (“inactivatable”) Xi on which loci have specific probabilities of being inactivated. Without allelic markers that would distinguish paternal and maternal Z/X chromosomes, we cannot determine whether silencing is random or imprinted. Allelic differences were used to demonstrate that both alleles are expressed, apparently at equivalent levels, in female platypus heterozygous for *FBX010*, *GLIS3*, and *SHB* [24]. These loci are all expressed from Xi in about half the cells, so the approximately equal representation of the two alleles might favor a random inactivation hypothesis; however peak intensity on sequencing trace files is at best semi-quantitative, so this question remains open.

### Ancient Gene Silencing and the Evolution of XCI

Because platypus sex chromosomes share homology with the chicken Z chromosome, our observation of inactivation in a proportion of cells for genes on the sex chromosomes in the homogametic sex of a representative bird (ZZ male chicken) and monotreme (XX female platypus) could be interpreted as the evolution of a silencing mechanism on an ancient bird-like Z in a common reptile-mammal ancestor. However, it is uncertain whether the bird Z/platypus X homology represents identity by descent (reviewed in [40]–[42]), but under either scenario the sex chromosome inactivation observed here is likely independently evolved [43].

Similar patterns of 1X gene expression in a proportion of cells have been observed in marsupials (tammar wallaby) [18] and escaper genes on the X in eutherians (elephant, mouse and human) [19]. The marsupial and eutherian X shares a large conserved region, which is completely non-homologous with the sex chromosomes of birds and monotremes. Thus, the probabilistic inactivation we observed in birds, monotremes and therian mammals was independently exapted from an ancient toolkit of mechanisms to turn off transcription of one allele. Indeed, this partial silencing system shares characteristics with monoallelic expression of some autosomal mammalian genes, such as interleukins (*IL2*, *IL4*, *IL5*, *IL10*, *IL13*) [44]–[48].

We propose that probabilistic silencing of the Z or X chromosome in the homogametic sex is an early step in the evolution of sex chromosome inactivation. A major difference in the inactivation systems is the extent of coordination. This is most evident for X inactivation in eutherian mammals, which is coordinately controlled by *XIST*, a locus that is absent outside eutherians [49]. In both chicken and platypus, the co-expression of neighbouring loci on the Za/X_5_a implies that the choice of which Zi/Xi to inactivate is controlled at least at the regional level, and possibly at the level of the whole chromosome, and in platypus (but not chicken) the probability of expression of neighbouring loci on X_5_i is at least regionally coordinated.

In eutherian mammals silencing was augmented by additional molecular changes (including histone modification and DNA methylation), into the stable and complex inactivation system typical of most genes on the conserved region of the eutherian X (XCR), which shows tight 1X-active expression observed at the single cell, as well as the population level [9], [19]. In contrast, the evolutionarily younger X added region of the human X (XAR) contains many escaper genes that show a probabilistic expression pattern similar to that of birds and monotremes. The marsupial X, though homologous to the eutherian XCR, displays a pattern of expression similar to the eutherian XAR, monotreme Xs and bird Z, suggesting that changes to the regulation of the eutherian XCR occurred after the divergence of marsupials from eutherian mammals 150 million years ago. This is consistent with the different epigenetic profiles displayed by the marsupial X and the eutherian XCR [15], [20], [50].

### Inactivation of Pseudoautosomal Genes

PAR inactivation has intriguing implications for sex chromosome evolution. The accepted hypothesis for the evolution of sex chromosomes and dosage compensation is that Y degeneration resulted in loss of Y gene function, which in turn drove the evolution of dosage compensation (resulting in XCI in therian mammals) [1], [51]. However, an alternate hypothesis is that the spread of the XCI signal into undifferentiated X regions preceded, and drove, degradation of homologous Y regions [52].

Genes in the human and mouse PAR are exempt from X-inactivation because the Y copy complements the X [53]. We therefore expected X and Y alleles of platypus PAR genes to be active in all nuclei in both sexes, as they are for genes in the human PAR1. Surprisingly, we observed a significantly lower frequency of 2X-active cells in both sexes for seven of the nine PAR loci. This confirms the observation [24] that other PAR loci were 1-allele active in platypus, and implies that inactivation of PAR genes regularly occurs in platypus. This contrasts with the expression of all autosome loci from both alleles in nearly all cells.

In females, inactivation of genes on the X PAR might be achieved via spreading of inactivation from the X-specific regions, as has been observed in the recently evolved human PAR 2 [54], [55]. However, PAR inactivation in males cannot be the result of XCI spreading, instead Y PARs could be inactivated by their proximity to heterochromatin on the male-specific region of the Y. It is possible that upregulation of the active X chromosomes in platypus, which maintained parity with autosomal genes, also resulted in upregulation of genes in the PARs. To mitigate this there was selection for partial inactivation of Xi PAR genes in females, and Y PAR genes in males.

In conclusion, our studies of dosage compensation on independently evolved amniote sex chromosomes reveals common patterns of inactivation. This suggests that repressive molecular mechanisms were independently exapted to reduce the probability of transcription from one Z or X chromosome in the homogametic sex, in response to upregulation (perhaps of only a subset genes) in the heterogametic sex.

## Materials and Methods

### Ethics Statement

The study was approved, and all samples were collected and held under The Australian National University Animal Experimentation Ethics Committee proposal numbers R.CG.11.06 and R.CG.14.08.

### Identification of BAC Gene Content

Genes were chosen based on three criteria: 1) Widespread expression in other species to maximize the chance they were expressed in platypus and chicken fibroblast cells. 2) A BAC was preferentially chosen if it contained a single gene. 3) Loci were selected that were distributed at roughly even intervals along chicken Z and platypus X_5_. On some of the platypus X chromosomes loci selection was limited by the paucity of anchored BACs. BACs containing chicken and platypus genes were ordered from CHORI BACPAC Resources Centre (http://bacpac.chori.org/). Chicken BACs bearing genes of interest were chosen using the UCSC genome browser BAC track (http://genome.ucsc.edu/cgi-bin/hgGateway). Platypus BACs were from Veyrunes et al. [23]. Additional platypus BACs were identified by blasting the BAC end trace archive (http://www.ncbi.nlm.nih.gov/Traces/trace.cgi).

### Cell Culture and RNA-FISH on Chicken and Platypus Interphase Nuclei

Chicken fibroblast cell lines were established from 8-day-old chicks; metaphase spreads were karyotypically normal. Platypus fibroblast cell lines were established from adult wild animals that were karyotypically normal.

Platypus and chicken fibroblast cells were cultured at 32°C and 37°C respectively in 5.0% CO_2_ on gelatin-coated coverslips in 1∶1 AmnioMax C100 medium (Invitrogen)/DMEM 10% FCS to a density of 60 to 80%. RNA-FISH was carried out as previously described [18]. Hybridization of the probe to homologous DNA will not occur because there is no DNA denaturation step in the RNA-FISH protocol.

### RNA-FISH Scoring and Statistical Analysis

For chicken RNA-FISH experiments a BAC from chromosome 1, which contained the gene *GAPDH* (CH261-14L1), was used as the autosomal control. For platypus RNA-FISH experiments a BAC from chromosome 6, which contained *HPRT1* (OaBb_405M2; GenBank Accession No. AC148426), was the autosomal control. Only diploid nuclei (two signals from the autosomal BAC) were scored for the test gene (X/Z or pseudoautosomal).

For each test gene, 1X- or 1Z-active nuclei were observed as one signal, and 2X- or 2Z-active nuclei were observed as two signals. Hybridization efficiencies (p) were obtained from results in the heterogametic sex (in which one signal is expected in all nuclei). These were used to calculate the expected frequency of nuclei with two signals, one signal, and no signals in the homogametic sex using the formula p^2^+2pq+q^2^ = 1; where p^2^ is the expected frequency of nuclei with two signals, 2pq (q = 1–p) is the frequency of nuclei with one signal, and q^2^ is the frequency with no signal. *P*-values were calculated using a χ^2^ test with two degrees of freedom and Bonferroni correction was conducted.

For a more rigorous statistical analysis we removed no signal cells from the dataset, and calculated experimental error using missed hybridization events in our autosomal RNA-FISH experiments. In chicken we conducted 16 autosomal RNA-FISH experiments and scored a total of 1756 nuclei, each of which should produce 2 signals, for a total of 3512 signals. We observed a total of 12 nuclei with no signals and 16 nuclei with one signal, which is equal to 40 missed hybridisations out of 3512 (1.14%). Following a Poisson distribution, 95% confidence limits for 40 events gives a minimum of 28.58 and a maximum of 54.47. The upper value of the 95% confidence limits represents 1.55% experimental error (i.e. 54.47 out of 3512). For platypus, using exactly the same approach (18 autosomal RNA-FISH experiments, 1884 nuclei scored in total, 3768 expected signals, 69 missed hybridisations, upper 95% confidence interval of 87.32), maximum experimental error was estimated at 2.32%. All observed values were adjusted towards the expected values by 1.55% and 2.32% in chicken and platypus respectively. P-values were recalculated using a χ^2^ test with one degree of freedom. Significance remained (after Bonferroni correction) for all BACs. Taking into account an arbitrary conservative experimental error of 10% (±5%), and adjusting all the observed values by 5% towards the expected values still results in significant p-values.

### Transcript Abundance

Expression values were obtained for known genes on each BAC in chicken and platypus (from [21], [34]), and expression ratios calculated. If a BAC carried more than one gene, expression data from the gene that spanned the largest proportion of the BAC was used (*DMRT1/3* expression data was not included due to its involvement in sex determination). For chicken expression data was available for brain, cerebellum, heart, kidney and liver. Because no data were available for cultured fibroblasts, an average M∶F expression ratio was taken across all tissues to best control for tissue specific expression changes. For platypus, data were available for cultured fibroblast cells, which was used to calculate F∶M expression ratios because the present study was conducted in cultured fibroblasts.

These expression ratios were plotted against the percentage of 1Z/1X-active nuclei observed for the relevant BAC. If an RNA-FISH experiment was repeated for a BAC, an average observed percentage of 1Z/X activity was used. Scatterplots were drawn (Figure S3), and R^2^ and p-values were calculated in Microsoft Excel.

### Scoring Coordinated Transcription/Transcription Inhibition of Adjacent Genes

RNA-FISH signals co-locate in the nucleus when closely linked and transcribed from the same chromosome, whereas signals from genes transcribed from different chromosomes (or distantly linked on the same chromosome) are further apart. We only used pairs of genes physically close enough to each other to give unmistakable results.

To determine if the there was a single active Z (or X for platypus), all nuclei that were 1Z (or 1X) active for both loci in a pair were scored. Co-location of the two signals was interpreted as a single active Z (or X) in that nucleus. The expected frequency of nuclei that were 2Z (or 2X) active for both loci in a gene pair was the product of individual 2Z (or 2X) frequencies of each gene (from initial RNA-FISH results). To determine if inactivation of neighbouring gene pairs was coordinated, this frequency was compared to the observed frequency of nuclei that were 2Z (or 2X) active for both loci. P-values were calculated using a χ^2^ test with one degree of freedom and Bonferroni correction was conducted.

## Supporting Information

Figure S1 DAPI stained plane of female chicken fibroblasts demonstrating high hybridization efficiency with the BAC CH261-100P10 (gene *CRIM1*). Insets are high magnification images of each nucleus to display the signals. Signals in a different focal plane, and appear weak, are arrowed. A tetraploid nucleus is denoted with 4n.
(TIF)

Figure S2 RNA-FISH experiments on daughter cells where one is 1Z/X active, and the other is 2Z/2X-active. A) Expression of *BNC2* in male chicken daughter cells. B) Expression of *SEMA6A* in female platypus daughter cells.
(TIF)

Figure S3 **A**) Chicken male: female transcript abundance ratio plotted against percentage of nuclei in males with 1Z-activity. **B**) Platypus female: male transcript abundance ratio plotted against percentage of nuclei in females with 1X-activity.
(DOCX)

Figure S4 DAPI stained plane of female platypus fibroblasts demonstrating high hybridization efficiency with the BAC CH236-27K18 (gene *IGF1R*). Insets are high magnification images of each nucleus to display the signals. Signals in a different focal plane, and appear weak, are arrowed. A tetraploid nucleus is denoted with 4n.
(TIF)

Figure S5 Sex chromosome system in a male platypus. PARs are notated according to the sex chromosomes that bear them, in order X_1_/Y_1_, X_2_/Y_1_, X_2_/Y_2_, X_3_/Y_2_, X_3_/Y_3_, X_4_/Y_3_, X_4_/Y_4_, X_5_/Y_4_, X_5_/Y_5_. Pink represent homologous regions. Lines represent loci analyzed in this study and in Deakin et al. [24].
(TIF)

Table S1 RNA-FISH data for chicken loci.
(DOC)

Table S2 RNA-FISH data for platypus loci.
(DOC)

Table S3 RNA-FISH dataset for human loci orthologous to chicken Z/platypus X loci.
(DOCX)
