# Supplementary figures and images for: Independent Evolution of Transcriptional Inactivation on Sex Chromosomes in Birds and Mammals
Source: PLoS Genet. 2013 Jul 18;9(7):e1003635. doi: 10.1371/journal.pgen.1003635 (PMC3715422; doi:10.1371/journal.pgen.1003635)

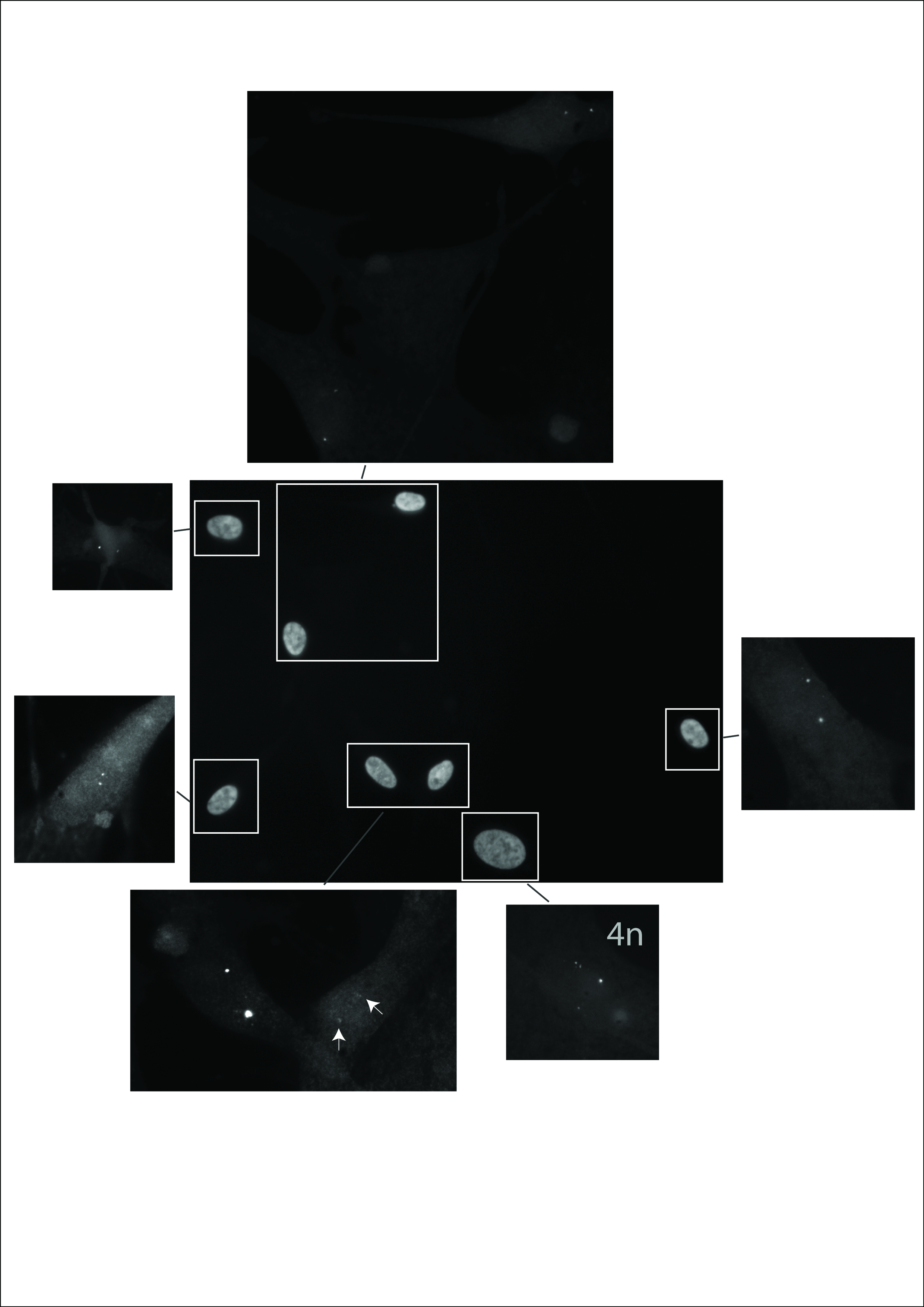

Supplement: Figure S1 — DAPI stained plane of female chicken fibroblasts demonstrating high hybridization efficiency with the BAC CH261-100P10 (gene CRIM1). Insets are high magnification images of each nucleus to display the signals. Signals in a different focal plane, and appear weak, are arrowed. A tetraploid nucleus is denoted with 4n. (TIF) [file pgen.1003635.s001.tif]

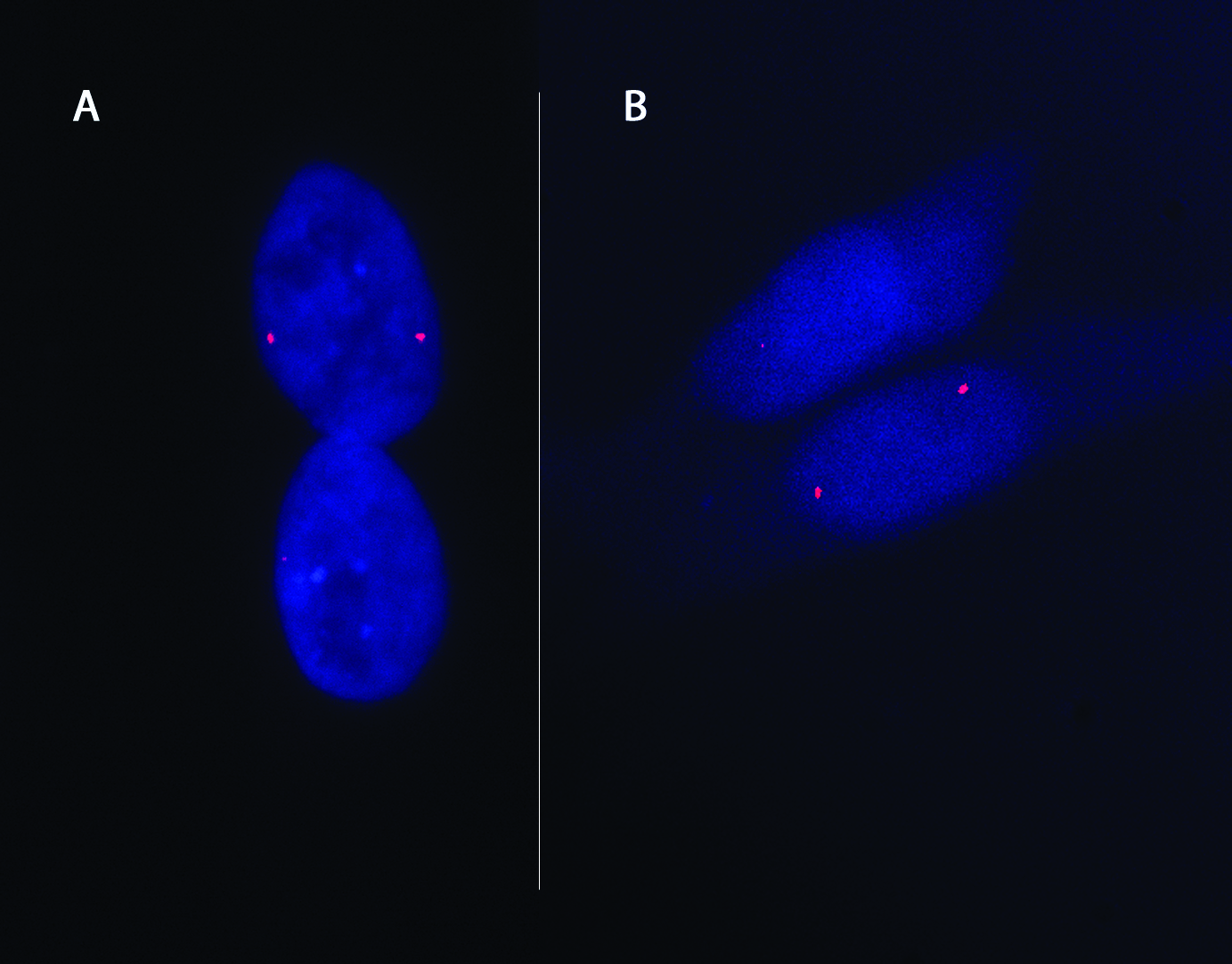

Supplement: Figure S2 — RNA-FISH experiments on daughter cells where one is 1Z/X active, and the other is 2Z/2X-active. A) Expression of BNC2 in male chicken daughter cells. B) Expression of SEMA6A in female platypus daughter cells. (TIF) [file pgen.1003635.s002.tif]

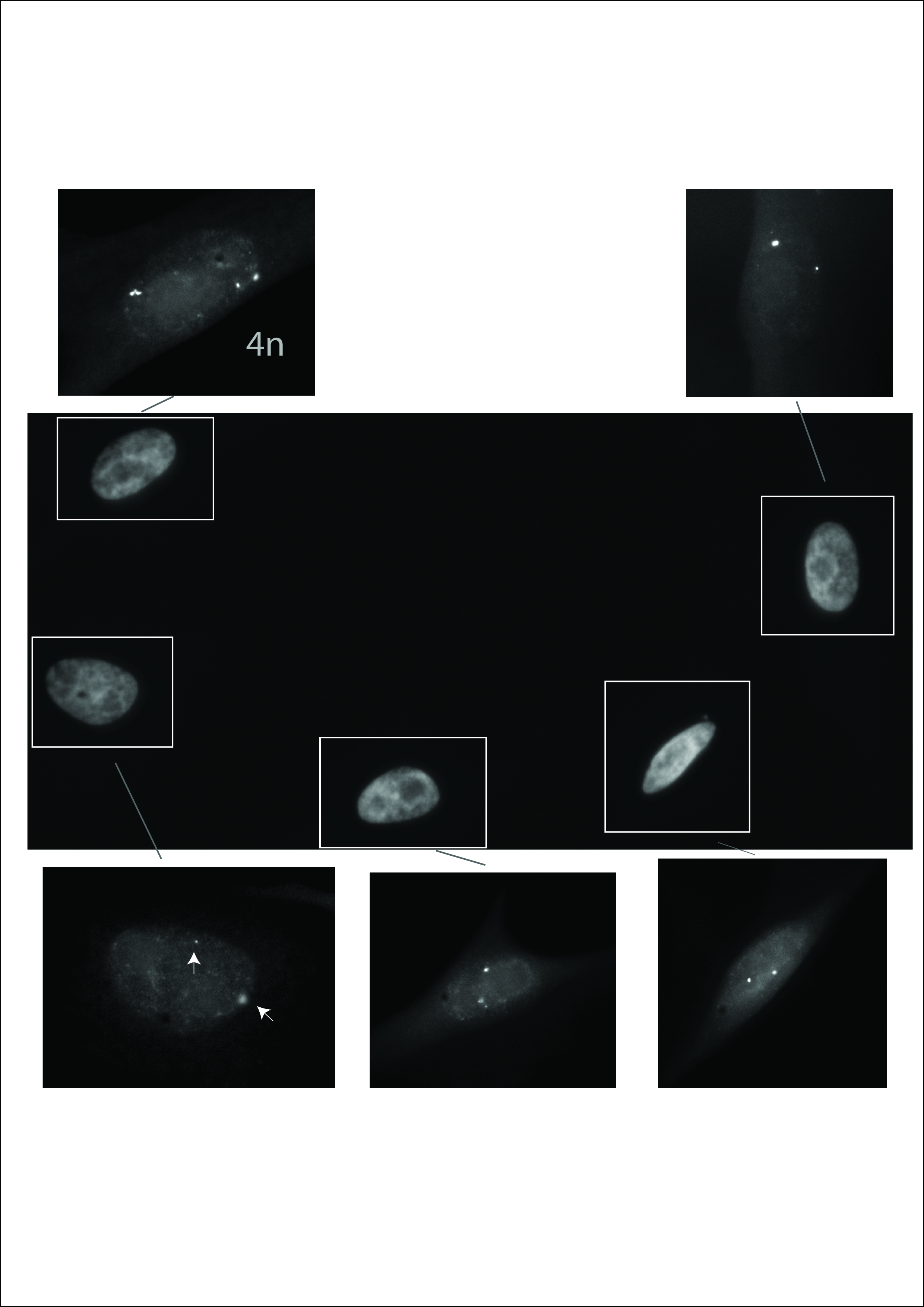

Supplement: Figure S4 — DAPI stained plane of female platypus fibroblasts demonstrating high hybridization efficiency with the BAC CH236-27K18 (gene IGF1R). Insets are high magnification images of each nucleus to display the signals. Signals in a different focal plane, and appear weak, are arrowed. A tetraploid nucleus is denoted with 4n. (TIF) [file pgen.1003635.s004.tif]

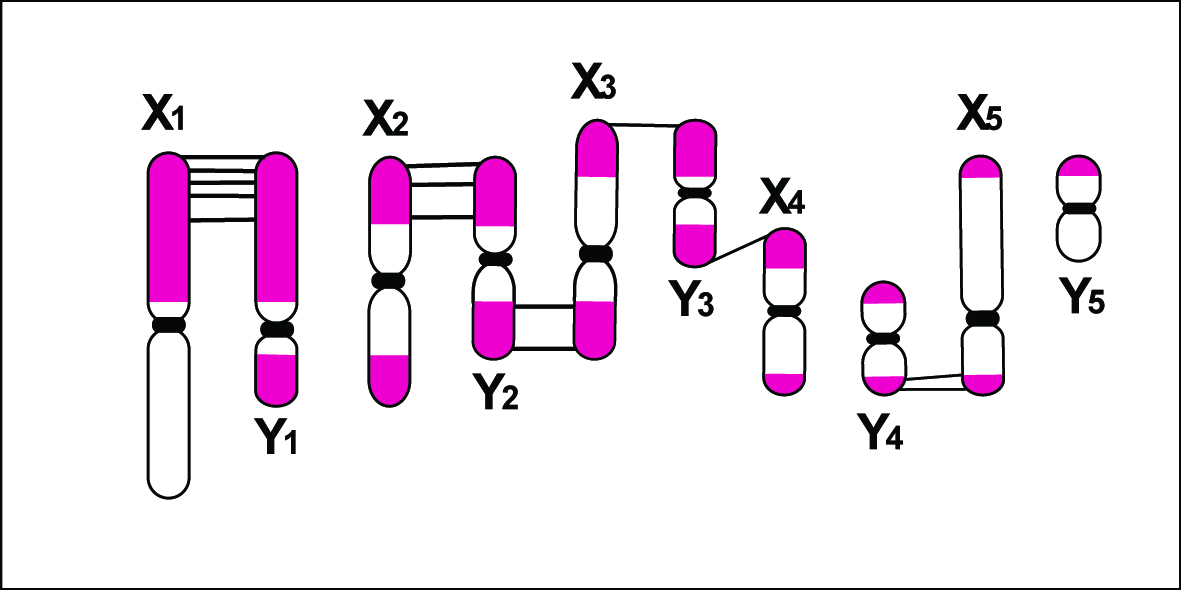

Supplement: Figure S5 — Sex chromosome system in a male platypus. PARs are notated according to the sex chromosomes that bear them, in order X1/Y1, X2/Y1, X2/Y2, X3/Y2, X3/Y3, X4/Y3, X4/Y4, X5/Y4, X5/Y5. Pink represent homologous regions. Lines represent loci analyzed in this study and in Deakin et al. [24]. (TIF) [file pgen.1003635.s005.tif]
