# Supplementary material for: Independent Evolution of Transcriptional Inactivation on Sex Chromosomes in Birds and Mammals
Source: PLoS Genet. 2013 Jul 18;9(7):e1003635. doi: 10.1371/journal.pgen.1003635 (PMC3715422; doi:10.1371/journal.pgen.1003635)
Supplement: Figure S3 — A) Chicken male: female transcript abundance ratio plotted against percentage of nuclei in males with 1Z-activity. B) Platypus female: male transcript abundance ratio plotted against percentage of nuclei in females with 1X-activity. (DOCX) [file pgen.1003635.s003.docx]

Figure S3a

Figure S3b
