# Supplementary material for: Independent Evolution of Transcriptional Inactivation on Sex Chromosomes in Birds and Mammals
Source: PLoS Genet. 2013 Jul 18;9(7):e1003635. doi: 10.1371/journal.pgen.1003635 (PMC3715422; doi:10.1371/journal.pgen.1003635)
Supplement: Table S1 — RNA-FISH data for chicken loci. (DOC) [file pgen.1003635.s006.doc]

**Table S1. RNA-FISH data for chicken loci.**

| **FISH location** | **BAC no.** | **Genes on BACs** | **Efficiency** | **Expected % male nuclei with 2 signals** | **Number of nuclei scored** | **Observed % nuclei with:** | | | **p-value** |
| --- | --- | --- | --- | --- | --- | --- | --- | --- | --- |
|  |  |  |  |  |  | **2 signals** | **1 signal** | **0 signals** |  |
| **Autosomal BACs** | | | | | | | | | |
| Chr 1 | CH261-14L1 | GAPDH + 10 more | NA | | 100^M^ | 97 | 0 | 3 | NA |
|  |  |  | NA | | 124^M^ | 97 | 2 | 1 | NA |
|  |  |  | NA | | 101^F^ | 99 | 0 | 2 | NA |
| Chr 2 | Ch261-15B20 | DPP6 | NA | | 101^M^ | 99 | 0 | 1 | NA |
|  |  |  | NA | | 104^F^ | 100 | 0 | 0 | NA |
| Chr 4 | Ch261-88M22^#^ | RBMX, RBMXL1 | NA | | 109^M^ | 99 | 0 | 1 | NA |
|  |  |  | NA | | 55^F^ | 100 | 0 | 0 | NA |
| Chr 4 | Ch261-41B17 | HS6ST1 | NA | | 100^M^ | 100 | 0 | 0 | NA |
|  |  |  | NA | | 111^F^ | 100 | 0 | 0 | NA |
| Chr 4 | Ch261-189A7^#^ | DIAPH2 | NA | | 100^M^ | 100 | 0 | 0 | NA |
|  |  |  | NA | | 128^F^ | 100 | 0 | 0 | NA |
| Chr 3 | CH261-100P10* | *CRIM1* | NA | | 134^F^ | 99 | 1 | 0 | NA |
| Chr 5 | CH261-21M6* | *EBF1* | NA | | 148^F^ | 98 | 1 | 1 | NA |
| Chr 1 | CH261-52K7 | *CAND1* | NA | | 105^F^ | 97 | 3 | 0 | NA |
| Chr 5 | CH261-64A1 | *GALNTL4* | NA | | 126^F^ | 96 | 4 | 0 | NA |
| Chr 5 | CH261-66B2 | *SOX6* | NA | | 110^F^ | 95 | 2.5 | 2.5 | NA |
| **Z-Specific BACs** | | | | | | | | | |
| Chr Z | Ch261-125B9 | *DMTR1/3, ANKRD15* | 95 | 90.25 | 123 | 84 | 15 | 1 | 0.027 |
| Chr Z | Ch261-110A9 | ***SMARCA2, Cr595322*** | 97 | 94.09 | 113 | 76 | 24 | 0 | <0.01 |
| Chr Z | Ch261-164N4 | ***PTPRD*** | 98 | 96.04 | 110 | 60 | 36 | 4 | <0.01 |
| Chr Z | Ch261-87K13 | *PSIP1, HDGF2* | 97 | 94.09 | 128 | 77 | 18 | 5 | <0.01 |
|  |  |  |  |  | 114 | 67 | *26* | 7 | <0.01 |
| Chr Z | Ch261-57B23 | ***BNC2*** | 95 | 90.25 | 113 | 73 | 27 | 0 | <0.01 |
| Chr Z | Ch261-89C2 | ***MLLT3, KIAA1797*** | 98 | 96.04 | 118 | 77 | 21 | 2 | <0.01 |
| Chr Z | Ch261-65D18 | *SLC12A1/2, FBN2* | 96 | 92.16 | 122 | 75 | 25 | 0 | <0.01 |
| Chr Z | Ch261-30H20 | *RASA1, CCNH* | 100 | 100.00 | 104 | 48 | 51 | 1 | <0.01 |
| Chr Z | Ch261-73F14 | *LINGO2* | 95 | 90.25 | 113 | 47 | 45 | 8 | <0.01 |
| Chr Z | Ch261-112C1 | *ACO1* | 96 | 92.16 | 113 | 58 | 40 | 2 | <0.01 |
| Chr Z | Ch261-163I20 | *HSD17B4* | 96 | 92.16 | 116 | 57 | 40 | 3 | <0.01 |

Nuclei that did not have two signals for the autosomal control were not scored for the Z locus. Please see Materials and Methods for description of RNA-FISH scoring. Genes names in bold were used for neighboring gene experiments. P-values were calculated with a X^2^ test with 1 degrees of freedom. Bonferroni correction was conducted. Superscript M and F denotes male and female derived cell lines. Numbers for Z specific loci were all generated from male cells. BAC names with a hash (#) are orthologous to human X genes. BAC names with an asterix (*) are orthologous to the platypus X_1_Y_1_ PAR.
