# Supplementary material for: Independent Evolution of Transcriptional Inactivation on Sex Chromosomes in Birds and Mammals
Source: PLoS Genet. 2013 Jul 18;9(7):e1003635. doi: 10.1371/journal.pgen.1003635 (PMC3715422; doi:10.1371/journal.pgen.1003635)
Supplement: Table S2 — RNA-FISH data for platypus loci. (DOC) [file pgen.1003635.s007.doc]

**Table S2. RNA-FISH data for platypus loci.**

| **FISH location** | **BAC no.** | **Genes on BACs** | **Efficiency** | **Expected % female nuclei with 2 signals** | **Number of nuclei scored** | **Observed % nuclei with:** | | | **P-value** |
| --- | --- | --- | --- | --- | --- | --- | --- | --- | --- |
|  |  |  |  |  |  | **2 signals** | **1 signal** | **0 signals** |  |
| **Autosomal BACs** | | | | | | | | | |
| Chr 6 | CH236-405M2^*#^ | *HPRT1* | N/A | | 86^F^ | 96.5 | 3.5 | 0 | N/A |
|  |  |  | N/A | | 89^M^ | 95.5 | 4.5 | 0 | N/A |
| Chr 6 | CH236-481H12^#^ | *CACNA1F, SYP, GPKOW, WDR45, OTUD5, MITF, KCNDI, PLP2, JM11* | N/A | | 100^F^ | 100 | 0 | 0 | N/A |
|  |  |  | N/A | | 105^M^ | 95 | 0 | 5 | N/A |
| Chr 6 | CH236-17J16^#^ | *BGN* | N/A | | 103^F^ | 97 | 1 | 2 | N/A |
|  |  |  | N/A | | 105^M^ | 95 | 2 | 3 | N/A |
| Chr 1 | CH236-359L11^#^ | *TBL1X* | N/A | | 108^F^ | 95 | 4 | 1 | N/A |
|  |  |  | N/A | | 105^M^ | 95 | 0 | 5 | N/A |
| Chr 18 | CH236-558D19 | *ST3GAL3, JMJD2A* | N/A | | 104^F^ | 96 | 3 | 1 | N/A |
|  |  |  | N/A | | 101^M^ | 99 | 0 | 1 | N/A |
| Chr 18 | CH236-427K7 | *TMEM131, VWA3B* | N/A | | 100^F^ | 100 | 0 | 0 | N/A |
|  |  |  | N/A | | 100^M^ | 100 | 0 | 0 | N/A |
| Chr 5 | CH236—27K18 | *IGF1R* | N/A | | 108^F^ | 97 | 3 | 0 | N/A |
| Chr 5 | CH236-165G18 | *TJP1* | N/A | | 100^F^ | 100 | 0 | 0 | N/A |
| Chr 10 | CH236-309G13 | *MKLN1* | N/A | | 102^F^ | 98 | 2 | 0 | N/A |
| Chr 12 | CH236-326M8 | *GPM6A* | N/A | | 124^F^ | 97 | 3 | 0 | N/A |
| Chr 18 | CH236-160D2 | *GPR125* | N/A | | 114^F^ | 97 | 3 | 0 | N/A |
| Chr 18 | CH236-46I13 | *RBPSUH* | N/A | | 129^F^ | 98 | 2 | 0 | N/A |
| **Pseudoautosomal** | | | | | | | | | |
| X_1_/Y_1_ | CH236-156C11 | *PDE6A,* | N/A | | 101^F^ | 65 | 27 | 8 | <0.01 |
|  |  |  |  | | 116^F^ | 48 | 47 | 5 | <0.01 |
|  |  |  |  | | 117^M^ | 53 | 42 | 5 | <0.01 |
|  | CH236-755N9 | unknown |  | | 102^F^ | 75 | 21 | 4 | >0.05 |
|  |  |  |  | | 102^M^ | 70 | 28 | 2 | <0.05 |
|  | CH236-40E 6 | *EBF1* |  | | 103^F^ | 83 | 16 | 1 | >0.05 |
|  |  |  |  | | 107^M^ | 75 | 24 | 1 | >0.05 |
| X_2_/Y_2_ | CH236-78K11 | *ZNF236* |  | | 101^F^ | 52 | 43 | 5 | <0.01 |
|  |  |  |  | | 111^F^ | 63 | 35 | 2 | <0.01 |
|  |  |  |  | | 100^M^ | 53 | 41 | 6 | <0.01 |
| X_3_/Y_2_ | CH236-50B13 | *MYO10* |  | | 106^M^ | 68 | 28 | 4 | <0.01 |
|  |  |  |  | | 114^M^ | 66 | 31 | 3 | <0.01 |
|  |  |  |  | | 117^F^ | 53 | 41 | 6 | <0.01 |
|  |  |  |  | | 126^F^ | 75 | 24 | 1 | <0.05 |
| X_3_/Y_3_ | Oa-Bb-462C1 | *unknown* |  | | 111^F^ | 70 | 25 | 5 | <0.01 |
|  |  |  |  | | 107^M^ | 68 | 28 | 4 | <0.01 |
| X_4_/Y_3_ | CH236-639O23 | *CACNAIB, EHMT1* |  | | 104^F^ | 67 | 30 | 3 | <0.01 |
|  |  |  |  | | 103^M^ | 58 | 40 | 2 | <0.01 |
| X_5_/Y_4_ | CH236-730N11 | *AGPAT1, EGFL8* |  | | 106^F^ | 68 | 29 | 3 | <0.01 |
|  |  |  |  | | 114^M^ | 67 | 31 | 2 | <0.01 |
|  | Oa-Bb-466A15 | *unknown* |  | | 100^F^ | 68 | 26 | 6 | <0.01 |
|  |  |  |  | | 100^M^ | 70 | 27 | 3 | <0.05 |
| **X-specific** | | | | | | | | | |
| X_1_ | CH236-797P4 | *PRICLE2* | 94 | 88 | 109 | 58 | 39 | 3 | <0.01 |
|  | CH236-40J11 | *FOXP1* | 95 | 90 | 117 | 32 | 62 | 6 | <0.01 |
| X_2_ | CH236-308M7 | *MLLT3* | 95 | 90 | 108 | 38 | 58 | 4 | <0.01 |
| X_3_ | CH236-24P4 | *PALM2* | 94 | 88 | 104 | 62 | 37 | 1 | <0.01 |
|  | CH236-81G18 | *NDUFS6, MRPL36* | 98 | 96 | 108 | 41 | 56 | 3 | <0.01 |
| X_5_ | CH236-246D1 | *RFX3* | 95 | 90 | 106 | 45 | 51 | 4 | <0.01 |
|  | CH236-7F2 | *SMARCA2* | 97 | 94 | 100 | 42 | 55 | 3 | <0.01 |
|  | CH236-257N3 | *ANKRD15* | 94 | 88 | 101 | 46 | 50 | 4 | <0.01 |
|  | CH236-34D10 | *RNF20* | 99 | 98 | 100 | 50 | 45 | 5 | <0.01 |
|  | CH236-38O6 | *PRR16* | 96 | 92 | 108 | 42 | 47 | 11 | <0.01 |
|  | CH236-408I6 | ***HSD17B4*** | 95 | 90 | 100 | 57 | 43 | 0 | <0.01 |
|  |  |  |  |  | 114 | 71 | 27 | 2 | <0.01 |
|  | CH236-752F12* | ***SEMA6A*** | 99 | 98 | 65 | 20 | 74 | 6 | <0.01 |
|  | CH236-271G4* | ***SLCIA1*** | 99 | 98 | 77 | 39 | 61 | 0 | <0.01 |
|  | CH236-6F5 | *TYRP1* | 96 | 92 | 103 | 50 | 47 | 3 | <0.01 |
|  |  |  |  |  | 101 | 62 | 32 | 6 | <0.01 |
|  | CH236-370K10 | ***MPDZ*** | 96 | 92 | 101 | 59 | 38 | 3 | <0.01 |
|  | CH236-334H12 | ***NFIB*** | 95 | 90 | 104 | 31 | 62 | 7 | <0.01 |
|  | CH236-221H7 | *PSIP1* | 99 | 98 | 104 | 46 | 52 | 2 | <0.01 |
|  |  |  |  |  | 102 | 69 | 29 | 2 | <0.01 |
|  | CH236-279E 13 | *BNC2* | 97 | 94 | 120 | 89 | 10 | 1 | <0.01 |
|  |  |  |  |  | 183 | 95 | 4 | 1 | <0.01 |
|  | CH236-197K12 | *ACO1* | 94 | 88 | 106 | 57 | 39 | 4 | <0.01 |
|  | CH236-63M3 | *LINGO2* | 97 | 94 | 101 | 69 | 26 | 5 | <0.01 |
|  |  |  |  |  | 103 | 61 | 39 | 0 | <0.01 |
|  | CH236-339J17 | *PLAA* | 99 | 98 | 106 | 66 | 25 | 9 | <0.01 |

Nuclei that did not have two signals for the autosomal control were not scored for the X locus. Please see Materials and Methods for description of RNA-FISH scoring. Genes names in bold were used for neighboring gene experiments. P-values were calculated with a X^2^ test with 1 degree of freedom. Bonferroni correction was conducted. Superscript M and F denotes male and female derived cell lines. Numbers for X specific loci were all generated from female cells. BAC names with a hash (#) are orthologous to human X genes. BAC names with an asterix (*) were also examined in reference [22].
