# Supplementary material for: Independent Evolution of Transcriptional Inactivation on Sex Chromosomes in Birds and Mammals
Source: PLoS Genet. 2013 Jul 18;9(7):e1003635. doi: 10.1371/journal.pgen.1003635 (PMC3715422; doi:10.1371/journal.pgen.1003635)
Supplement: Table S3 — RNA-FISH dataset for human loci orthologous to chicken Z/platypus X loci. (DOCX) [file pgen.1003635.s008.docx]

**Table S3. RNA-FISH dataset for Human loci orthologous to chicken Z/platypus X loci.**

| **FISH location** | **BAC no.** | **Genes on BACs** | **Number of nuclei scored** | **Observed % nuclei with** | | |
| --- | --- | --- | --- | --- | --- | --- |
|  |  |  |  | **2 signals** | **1 signal** | **0 signals** |
| Chr 9 | RP11-641M16 | *LINGO2* | 101^M^ | 94 | 3 | 3 |
| Chr 9 | RP11-794C19 | *ACO1* | 102^F^ | 96 | 2 | 2 |
| Chr 5 | RP11-632B20 | *HSD17B4* | 111^F^ | 96 | 3 | 1 |
| Chr 9 | RP11-938H15 | *BNC2* | 144^F^ | 98 | 2 | 0 |

All RNA-FISH experiments were done in conjunction with an autosomal control BAC RP11-63K19 containing *GBA*. Superscript M and F denotes male and female derived cell lines. All nuclei that did not have two signals for the control BAC were removed from the dataset.
